# Beef flavor vegetable hamburger patties with high moisture meat analogs (HMMA) with pulse proteins‐peas, lentils, and faba beans

**DOI:** 10.1002/fsn3.2172

**Published:** 2021-05-17

**Authors:** Taehoon Kim, Rhonda Miller, Hannah Laird, Mian N. Riaz

**Affiliations:** ^1^ Department of Nutrition and Food Science Texas A&M University College Station TX USA; ^2^ Department of Animal Science Texas A&M University College Station TX USA; ^3^ Food Science and Technology Dept Texas A&M University College Station TX USA

## Abstract

Pulses have been an excellence source of foods due to their nutritional profile including high protein content. In addition, they have less concerns about allergens, gluten, and genetically modified organisms. In this study, high moisture meat analogs (HMMA) that contained commercial pea protein (55.4% protein), lentil protein (55.4% protein), or faba bean protein (61.5% protein) mixed with other constant ingredients (pea isolates, and wheat gluten and canola oil) were produced using a twin‐screw extruder (TX‐52) with an attached cooling chamber. For consumer sensory tests and texture profile analysis, vegetable hamburger patties with HMMA were produced with the addition of spices, binders, and so on. Trained panelists reported that HMMA with pulses had higher scores on bean‐like, sweet, and cohesiveness of mass compared to HMMA with soy that had higher soy, cardboardy, hardness, and springiness scores. Compared to the control, consumer panelists indicated that samples containing pulse proteins had no differences in consumers’ liking for the cooked appearance and overall flavor, but had a lower overall texture liking score, and samples with faba bean proteins (FP) had a lower overall liking score. Cooked patties containing pulse proteins were redder and had more cooking yield. Patties with FP required less cooking time. Therefore, vegetable patties with pulse proteins are competitive with soy‐based samples.

## Graphical abstract

Consumer like (a) or dislike (b) descriptors for vegetable patties containing high moisture meat analog with lentil proteins.

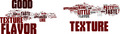

## 1 INTRODUCTION

High moisture meat analogs (HMMA) are protein products produced by an extrusion process with the addition of moisture (40% to 80%) during the process to prevent expansion of the product in a cooling die attached to the end of the extruder. Unlike low moisture extruded protein products, HMMAs have well defined fiber formations, resemble chicken or turkey breast meat, and therefore have an enhanced visual appearance and taste sensation s (Malav et al., 2015; Sadler, 2004). These meat analogs are also called meat substitutes, mock meat, faux meat, or imitation meat (Sadler, 2004). The key ingredients used during the preparation of meat analogs are soy protein, mushrooms, wheat gluten, egg albumin, carbohydrates, gum, and flavoring and other miscellaneous compounds such as fiber, caseinate, or carrageenan, as needed (Kumar et al., 2017).

Pulses are the dry edible seeds of plants in the legume family including field peas, dry beans, lentils, chickpeas, and faba beans (Tyler et al., 2017). Pulses have a high protein content (about 20%–40%) and are abundant in dietary fiber, resistant starch, vitamins, and minerals (Sozer et al., 2017). In addition, the Frost and Sullivan Analysis found that pulses are considered nonallergenic, non‐GMO, and appeal to vegans (Crane, 2015). Therefore, pulses can be excellent alternative meat sources.

The objectives of this study were to understand the trained panelist's perceptions of HMMA, the consumers’ perceptions of vegetable hamburger patties with HMMA formulated with the addition of spices, binders, and so on, and the relationships between the trained panelists’ tests and the consumers’ tests. The hypothesis was that the qualities of the HMMA used in the trained panelists’ tests and the consumers’ tests would not be significantly different from the control (soy), and both tests would have a strong relationship in evaluating the quality of the products. Therefore, this study will contribute to developing a vegetable patty as a meat substitute, for which the consumer demand is rising steadily worldwide.

## 2 MATERIALS AND METHODS

### 2.1 Trained panelist test

#### 2.1.1 Sample preparations

The HMMA samples produced in the research (Kim, Riaz, Awika, & Teferra, 2021) were used in this research. The HMMA samples are identified as C1 with soy concentrate (control), T1 with pea protein, T2 with lentil protein, and T3 with faba bean protein. The recipe, C1, containing soy concentrate and soy isolates and constant ingredients (6% canola oil and 15% wheat gluten) served as a control. There recipes (T1, T2, and T3) with pea isolates and pulse protein were mixed with the constant ingredients (6% canola oil and 15% wheat gluten) for the HMMA. Frozen HMMA (C1, T1, T2, and T3) were thawed in the refrigeration for 24 hrs. Samples for each treatment were randomly selected. Before being served to trained panelists, they were boiled for 2 mins and stored in an oven at 80ºC covered with aluminum foil. The samples in the oven were cut into 2 cm cubes and placed in randomized plastic cups to be served.

#### 2.1.2 Sensory evaluation with trained panelists

The treated samples were evaluated by nine trained panelists from Texas A&M University who have been trained to evaluate beef flavor descriptive attributes. They were also trained for 3 days to help them become familiar and understand the attributes of a vegetable patty. Panelist training and testing was approved by the Institutional Review Board for the Protection of Human Subjects in Research (IRB) protocol IRB2017‐0362 M. Each trained panelist had a packet including the attributes of the food, double‐distilled deionized water, sparkling water, and saltless saltines. On the first day, panelists learned about basic tastes, cardboardy, grainy, musty‐earth/hummus, malt‐like, hay‐like, buttery, and heated oil. On the second day, the panelists learned about greens, lentils, vegetable IDs, celery, carrots, roots, starches, faba beans, peas, and soy. Next day, the panelists learned texture including cohesiveness, hardness, springiness, particle size, and slipperiness. On the last day, the training on the third day will be repeated to help in understanding. Screened lexicons and remaining lexicons such as flavors (starchy, grainy, bean‐like, soy‐like, green, salty, sweet, umami, cardboardy, musty‐earthy, malt‐like, buttery, heated oil, cohesiveness of mass (COM)) and texture attributes (hardness and springiness) were tested with trained panelists. At the end of each training day, each sample in a randomized plastic cube was given to the trained panelists to determine the appropriate lexicons applicable to describe the characteristics of the samples.

Panelists received a warm‐up sample to calibrate each sensory day, and the warm‐up was individually evaluated by each panelist and discussed. Panelists came to consensus for all attributes prior to testing. Each sample was served in a plastic cup marked with a random three‐digit code. Double‐distilled deionized water was prepared as a mouth cleanser between samples. Each panelist was given a tablet (iPad Air 1, Apple Inc., Cupertino, CA) to record their individual data using an electronic spreadsheet (Microsoft Excel, One Drive, Microsoft Corporation, Redmond, WA), and samples were evaluated independently. Four random samples over the course of a two‐hour session were evaluated each sensory day.

### 2.2 Consumer test

#### 2.2.1 Materials

Commercially available, minced dried onion and black pepper (Member's Mark Minced Onion by Tone's, ACH Food Companies, Inc., Memphis, TN), lactic acid (Druids Grove Lactic Acid, Modernist Pantry LLC, Eliot, ME), and citric acid (Millard Citric Acid, Millard Brands, Lakewood, NJ) were purchased. Nonwhipping egg white power (Spray Dried Standard Egg Whites) was obtained from Sonstegard Foods Co. (Sioux Falls, *SD*), and beef flavor (TasteEssentials™ Nat Beef Vegetarian Flavor Type) was obtained from Givaudan (Cincinnati, OH). Natural flavor enhancer was obtained from Kikkoman (San Francisco, CA), and methylcellulose (Methocel SG A16 M Food Grade Modified Cellulose) was obtained from The Dow Chemical Company (Midland, MI). Shortening (SanTrans™ 39) was obtained from Loders Corklaan USA, LLC (Channahon, IL).

Frozen HMMA samples were stored for 24 hrs in the refrigerator at 4ºC. The HMMA samples were boiled for 2 mins as was done in the trained panelists test, resized using a size reducer (Comitrol 3,500, Urschel Laboratories, Inc., Valparaiso, IN) with a 9‐mm blade, and stored in the refrigerator for further experiments. Table 1 shows the recipe used to produce a vegetable hamburger patty for the consumer test. The spice mixture including minced onion, dried egg white, beef flavor, carrageenan, and flavor enhancers was prepared in the mixer, and lactic acid and citric acid were mixed to form a homogenous dry ingredient mixture. Shortening was chilled, ground through a 3.18 mm grinder plate and frozen. The frozen strings were broken into fat pellets and stored in the freezer until they were added to the mixture to make a vegetable patty.

**TABLE 1:** Recipe for producing a vegetable hamburger patty with a high moisture meat analog

Ingredients	g/100g
HMMA	53.28
Chilled Water, g	28.61
Minced Dried Onion	0.90
Egg White Powder (nonwhipping)	5.39
Carrageenan (Kappa)	0.49
Beef Flavor	2.69
Black Pepper	0.20
Natural Flavor Enhancer	0.45
Lactic Acid	0.45
Citric Acid	0.05
Methylcellulose	1.24
Shortening	6.26
Total	100.00

#### 2.2.2 Making patties

For the consumer test, the HMMA (0‐2ºC) chilled with ice in a container were mixed in a Hobart mixer (Hobart mixer, Model N50, Canada) at 20 rpm controlled by a rheostat (Type 3PN1010, Staco Energy Products Co., Dayton, OH) as water (0‐2ºC) chilled with ice in a container was added slowly. During mixing, methylcellulose was sprinkled in slowly for long enough to ensure a uniform methylcellulose coating of the HMMA particles. The dry ingredient mixture was added slowly to ensure uniform distribution of all ingredients followed by the addition of the shortening until the mix was uniform.

Patties for each treatment were formed with a patty maker (Supermodel 54 Food Portioning Machine, Hollymatic Corporation, Countryside, IL) with a 2.54 cm plate. The patties were placed with patty paper on top and bottom in a single layer on trays, placed in a −40℃ freezer, crust frozen for 20 mins, vacuum packaged, and stored in the −40℃ freezer until the sensory test.

#### 2.2.3 Cooking protocols

Approximately 24 hrs prior to testing, samples were removed from the freezer and placed on racks in a single layer to thaw in a cooler (4℃). One hour before testing, patties were organized by cooking order on the trays. Their vacuum packaged bags and patty paper were removed, and patty trays were covered with plastic wrap and held in the cooler until time to cook. Prior to cooking, five temperature readings of the surface of the grill were checked using an infrared temperature reader (MS6530H Infrared Thermometer, Commercial Electric Products Corporation, Cleveland, OH) with a target temperature of 162°C. As seen in APPENDIX A, the weights and temperatures of the raw samples and the time they were put on the grill were recorded, along with the end temperature, time they were taken off the grill, and final cooked weights.

Samples were cooked on a commercial flat‐top grill to an end temperature of 71°C, with a flip temperature at 27**°**C. Internal temperatures were monitored using thermocouple probes (Model SCPSS‐040 U‐6, Type T, 0.040 Sheath Diameter, 15.24 cm length Ungrounded Junction Thermocouple, Omega Engineering, Stamford, CT) by inserting them into the geometric center of each vegetable patty periodically during cooking. The temperature was displayed using a thermometer (Omega HH501BT Type T, Omega Engineering, Stanford, CT). Each sample was prepared on a clear plastic plate (clear 15.88 cm plastic plates premium quality, Members Mark, Sam's Club, Bentonville, AR) marked with a random three‐digit code. Each patty was cut into four equal pieces, and a quarter of a patty was served to each consumer. Consumers were given a new transparent plastic fork and transparent plastic knife to use for each sample as well.

After patties were taken off the grill and weighed, they were wrapped in foil and placed in a holding oven (Model 750‐TH‐II, Alto‐Shaam, Menomonee Falls, WI) for no longer than 20 mins, until served.

#### 2.2.4 Sensory test

In advance, 80 consumers were recruited by emails and advertisements. They provided demographic information and signed a consent form through a survey website (www.tamuag.az1.qualtrics.com ). Depending on their answers of available time for the test, they were assigned to one of four different sessions (20 consumer panelists each) for a 1 hr interval. In each session, they were divided into five groups since four wedges were cut from each patty. Four consumer panelists in each randomized group had the same treatment in the same order (APPENDIX A). Before the test, a consent form from each panelist was collected again. When they were seated in the booth under a red light, they were given a packet containing testing procedures, palate cleansers of distilled water and saltless saltine crackers, a demographic ballot, and five individual sample ballots. Demographic information from each panelist including gender, age, ethnicity, household income, household population, employment level, protein sources consumed, and location consumed, frequency of protein consumption, preferred cooking method for ground beef, degree of doneness desired for ground beef, type of ground beef typically purchased, desired fat percentage of ground beef, and types of cuisines consumed was received (APPENDIX B). Cooked appearance, overall appearance, overall flavor, and overall texture were evaluated by the panelists on each sample ballot utilizing a 9‐point hedonic scale. Open‐ended questions, “Please write any words that describe what you LIKE about this meat patty” and “Please write any words that describe what you DISLIKE about this meat patty,” were included on each ballot (APPENDIX C).

#### 2.2.5 Cooking yield and cooking time

Cooking yield was calculated by using Equation.1.

(1) $$\text{Cooking yield}\;\left(\%\right)=\frac{\text{Cooked patty weight}(g)}{\text{Raw patty weight}\left(g\right)}\times 100$$

Cooking time of each patty in minutes was measured.

#### 2.2.6 Color measurement

Frozen vegetable hamburger patties were thawed for 24‐hrs in a cooler (4°C) and remained at room temperature about 20 min after their vacuum packaged bags and patty paper were removed. Three locations on each patty were directly evaluated using a colorimeter (Model CR‐200, Minolta Co., Ramsey, NJ, USA). Values were expressed as L*, a*, and b*, where L* (lightness) vary from black (0) to white (100), chroma a* (redness) values vary from green (‐60) to red (+60), and chroma b* values (yellowness) vary from blue (‐60) to yellow (+60). Cooked hamburger patties were measured as well. Color measurements were made with three samples for each treatment.

#### 2.2.7 Texture analysis

A texture analysis of the vegetable hamburger patty was performed with a TA‐XT2 Texture Analyzer (Texture Technologies Corp., Scarsdale, NY) using the texture profile analysis measurement. According to the modified method of Ganhão et al. (2010), a cylindrical sample (2.54 cm diameter) from the center of each patty was sampled. A two‐cycle compression test was conducted to compress the sample to 70% of the original height with a cylindrical probe of 7.25 cm diameter and cross‐head speed of 1 mm/s. Texture profile parameters were evaluated following descriptions by Bourne (1978). All analyses were performed with five samples for each treatment.

### 2.3 Statistical Analysis

Data based on each protein type (C1, T1, T2, and T3) for each parameter were prepared for the statistical analysis of the data. The trained panel descriptive flavor and texture attributes, consumer preferences, color (raw and cooked), cooking yield, and cooking time of the samples were analyzed using the general linear mode procedure in SAS (9.4, SAS Institute, Cary, NC) with a predetermined alpha of 5%. For the trained panel results, data were averaged across panelists, order was defined as a random variable, and replicate was included in the model as a fixed effect. A full model was calculated where main effect of protein types was included. A one‐way ANOVA was used to determine the significant difference (*p* < .05) between vegetable hamburger patties containing HMMA with different protein sources. Color (L*, a*, and b*) for raw and cooked, cooking yield, and cooking time of the beef hamburger patties data were analyzed similarly. Least square means were calculated, and differences between least squares means were determined using the pdiff function when differences were significance (*p* < .05) in the Analysis of Variance table.

## 3 RESULTS AND DISCUSSION

### 3.1 Trained Descriptive Flavor and Texture Perception

The trained panelists’ perceptions are reported in Table 2. Protein sources in vegetable patties containing HMMA did not significantly affect flavor attributes for starchy (*p* = .58), grainy (*p* = .87), green (*p* = .65), and buttery (*p* = .41). Trained panelists could not perceive green and buttery in all samples.

**TABLE 2:** Trained descriptive flavor and texture perception of vegetable patties containing HMMA with pulse proteins

Attribute	P‐value	C1	T1	T2	T3	^b^RMSE
Flavor						
Starchy	0.58	3.7	3.8	3.9	4.0	0.34
Grainy	0.87	3.0	3.1	3.1	3.1	0.4
Bean‐like	<0.0001	1.9^a^	3.1^b^	3.2^b^	3.5^b^	0.33
Soy	0.01	4.4^a^	3.7^b^	3.8^b^	3.6^b^	0.44
Green	0.65	0.0	0.0	0.0	0.0	0.05
Salty	<0.001	1.4^a^	2.5^b^	2.5^b^	2.6^b^	0.29
Sweet	<0.0001	1.3^a^	2.4^b^	2.3^b^, ^c^	2.2^c^	0.16
Umami	<0.0001	2.1^a^	3.9^b^	4.0^b^	3.9^b^	0.41
Cardboardy	<0.0001	3.8^a^	2.5^b^	2.6^b^	2.6^b^	0.36
Musty‐Earthy	0.0063	1.7^a^	2.0^b^	2.0^b^	2.1^b^	0.22
Malt‐Like	<0.0001	1.6^a^	2.4^b^	2.4^b^	2.2^b^	0.26
Buttery	0.41	0.0	0.0	0.0	0.0	0.02
Heated Oil	<0.0001	1.9^a^	2.6^b^	2.7^b^	2.8^b^	0.24
Texture						
Cohesiveness of Mass	<0.0001	4.0^a^	6.8^b^	7.0^b^	7.2^b^	0.74
Hardness	<0.0001	8.3^a^	4.7^b^	4.0^b^, ^c^	3.9^c^	0.67
Springiness	<0.0001	8.0^a^	4.9^b^	4.7^b^	4.3^b^	0.58

^a^ Means within a row and effect followed by the same letter are not significantly different (*p* >.05).

^b^ RMSE = root mean square error.

^c^ Recipes for texturization: C1 (control) = soy concentrate and soy isolate, T1, T2, and T3 = pea proteins, lentil proteins, and faba bean proteins, respectively, premixed with a constant ingredient (canola oil and wheat gluten).

Compared to the control, C1, the samples (T1, T2, and T3) containing pulse proteins were scored higher for flavor attributes that were bean‐like, salty, sweet, umami, musty‐earthy, and malt‐like and heated oil indicated extremely small values (*p* < .0001). In contrast, these samples were lower for soy (*p* = .01) and cardboardy (*p* < .0001). However, these pulses did not have significantly different flavor attributes from each other except the sweetness attribute for T1 was higher than T3.

Pulse proteins in vegetable patties containing HMMA significantly affected texture attributes of cohesiveness of mass, hardness, and springiness resulting in extremely small values (*p* < .0001) compared to C1. The samples including pules proteins (T1, T2, and T3) were significantly higher for cohesiveness of mass and lower for hardness and springiness compared to the control. T1 was higher for hardness compared to T3.

Pulse proteins in the samples did not affect the flavor including starchy, grain, green, and buttery attributes compared to the control.

### 3.2 Consumer Demographics

Demographic information for consumers (*n* = 80) participating in this study is reported in Table 3. More females (73.4%) participated in the study compared to males, and the majority of participants (67.5%) fell in the 20 years or younger age group, followed by 21 to 25 (26.3%). The rest of the age groups that participated in this study were in the 26–35 age range (3.8%) and 36–45 age range (2.5%). The majority of consumers represented the Caucasian (non‐Hispanic) ethnicity (54.4%), followed by Asian/Pacific Islanders (17.7%), Latinos or Hispanics (16.5%), and African‐Americans (6.3%). Household incomes were distributed with 29.1% falling into the below $25,000 group, 7.6% falling into the $25,001 ‐ $49,999 group, 12.7% falling into the $50,000 ‐ $74,999 group, and the $75,000 ‐ $99,999 group, and 30.4% falling in the $100,000 group. Household size was represented by a majority of four‐person households (41.3%), followed by three‐person (22.5%), five‐person (15.0%), and two‐person (11.3%) households. The majority of participants (60%) were not‐employed, followed by participants who were employed part‐time (36.3%).

**TABLE 3:** Demographic frequencies for vegetable patty consumers (*n* = 80)

Question	Number of Respondents	Percentage of Respondents
Sex		
Male	21	26.6
Female	58	73.4
Age		
20 years or younger	54	67.5
21 – 25 years	21	26.3
26 – 35 years	3	3.8
36 – 45 years	2	2.5
46 – 55 years	0	0
56 – 65 years	0	0
66 years and older	0	0
Ethnicity		
African‐American	5	6.3
Asian/Pacific Islanders	14	17.7
Caucasian (non‐Hispanic)	43	54.4
Latino or Hispanic	13	16.5
Native American	1	1.3
Other	3	3.8
Household income		
Below $25,000	23	29.1
$25,001 ‐ $49,999	6	7.6
$50,000 ‐ $74,999	10	12.7
$75,000 ‐ $99,999	16	20.3
$100,000 or more	24	30.4
Household size including yourself		
1	3	3.8
2	9	11.3
3	18	22.5
4	33	41.3
5	12	15.0
6 or more	5	6.3
Employment level		
Not employed	48	60.0
Part‐time	29	36.3
Full‐time	3	3.8

When asked about proteins consumed at home, over 80% of consumers reported consuming chicken, beef (steaks), ground beef, and eggs, followed by fish (79.7%) and pork (70.1%) (data not presented). The top two proteins consumed at home included eggs (98.6%) and chicken (92.5%). When asked about proteins consumed away from home or at restaurants, over 80% of consumers reported consuming chicken, beef (steaks), ground beef, fish, and eggs. The top proteins consumed away from home included chicken (98.6%), ground beef (95.7%), eggs (88.9%), and ground beef (87.0%).

Consumers were asked to report how many times a week they consumed each protein source (data not presented). The majority of consumers reported consuming beef (steaks) 1 to 2 times per week (60.5%), followed by 0 times per week (26.3%) and 3 to 4 times per week (10.5%). For ground beef consumption, the majority of consumers reported eating it 1 to 2 times per week (66.2%) followed by 0 times per week (18.2%) and 3 to 4 times per week (15.6%). For pork consumption, consumers reported eating it 1 to 2 times per week (56.2%) followed by 0 times per week (38.4%). For lamb consumption, the majority of consumers reported 0 times per week (92.8%) followed by 1 to 2 times (7.2%). For chicken consumption, the majority of consumers consumed chicken 3 to 4 times per week (50.0%), followed by 1 to 2 times per week (23.1%) and 5 to 6 times per week (21.8%). For fish consumption, the majority of consumers reported eating fish 1 to 2 time per week (64.5%) followed by zero times per week (27.6%). Finally, for soy‐based products, consumers reported eating soy‐based products 0 times per week (64.3%) followed by 1 to 2 times per week (21.4%).

Consumers were asked what methods were preferred when cooking ground beef (data not presented). The majority of consumers preferred to pan‐fry/skillet on the stove (77.5%). Some consumers grilled outside (45.0%) and oven baked (22.5%), and even fewer used stir frying (17.5%), an electric appliance (George Forman Grill; 15.0%), oven broiling (8.8%), or a microwave (6.3%).

When asked for preferences on degree of doneness, consumers reported fairly evenly distributed between medium rare to well (data not presented). They reported medium (26.3%), medium well (22.5%), and both medium rare (20%) and well done (20%). Few consumers preferred the extremes with only 2.5% reporting rare and 8.8% for very well done.

When consumers were asked what fat level they typically purchased, consumers responded with both 7% and 10% with a 20% fat level, followed by 4% (21.3%), 15% (16.3%), and 20% (7.5%) (data not presented).

Consumers were asked what types of cuisines they liked to purchase (data not presented). Over 80% reported enjoying American, Barbeque, Mexican/Spanish, and Italian cuisines, followed by Chinese (76.3%) and Japanese (62.5%). Lebanese (23.8%), Indian (33.8%), French (43.8%), and Greek (47.5%) were among the lowest typically consumed. These results indicate that consumers in this study were an acceptable population to test vegetable patties containing (C1, T1, T2, and T3).

### 3.3 Consumer perception of vegetable patties containing HMMA

Consumer perception scores are reported in Table 4. Protein sources in meat patties containing HMMA did not significantly affect the number of consumers who liked the cooked appearance (*p* = .89) and overall flavor (*p* = .24). However, C1 was more desirable for overall liking and overall texture than T3.

**TABLE 4:** Consumer liking for HMMA vegetable patties

Attribute	*P*‐value	C1	T1	T2	T3	^b^RMSE
Cooked appearance	0.89	5.5	5.2	5.4	5.3	2.05
Overall	0.11	6.0^a^	5.3^a^, ^b^	5.4^a^, ^b^	5.0^b^	2.03
Overall flavor	0.24	6.0	5.3	5.4	5.1	2.09
Overall texture	0.003	6.0^a^	4.9^b^	4.8^b^	4.7^b^	2.16

^a^ Means within a row and effect followed by the same letter are not significantly different (*p* >.05).

^b^ RMSE = root mean square error.

^c^ Recipes for texturization: C1 (control) = soy concentrate and soy isolate, T1, T2, and T3 = pea proteins, lentil proteins, and faba bean proteins, respectively, premixed with a constant ingredient (canola oil and wheat gluten).

^d^ Consumer likes were measured with 0 = extremely dislike and 9 = extremely like.

**FIGURE 1:**
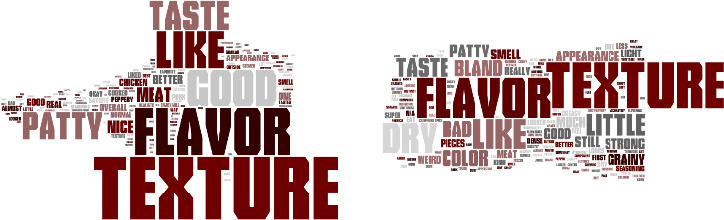
Consumer like (a) or dislike (b) descriptors for vegetable patties containing HMMA with C1

**FIGURE 2:**
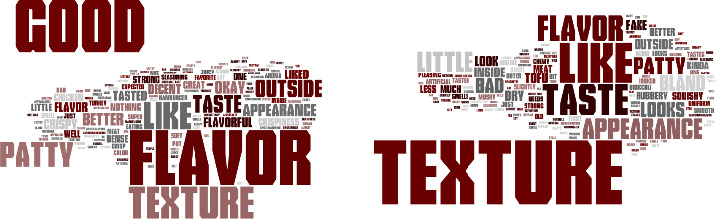
Consumer like (a) or dislike (b) descriptors for vegetable patties containing HMMA with T1

**FIGURE 3:**
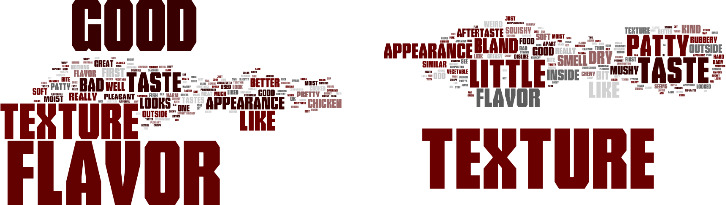
Consumer like (a) or dislike (b) descriptors for vegetable patties containing HMMA with T2

**FIGURE 4:**
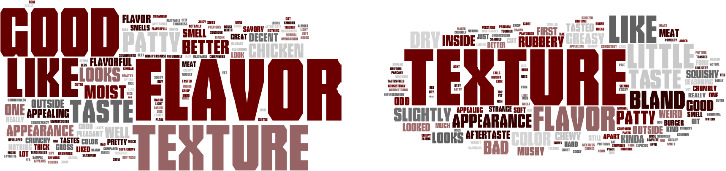
Consumer like (a) or dislike (b) descriptors for vegetable patties containing HMMA with T3

Word clouds were created using the comments from consumer panelists answers to an open‐ended question about whether or not they liked or disliked vegetable patties containing HMMA. Figure 1 to 4 demonstrate the consumer's responses separated by vegetable patties containing HMMA with different protein sources (C1, T1, T2, and T3). The size of the word illustrates how often the consumers used the words. For C1, the most commonly used words for liking were texture, flavor, good, like, and taste (Figure 1) and for disliking most commonly used words were texture, flavor, bad, little, dry, and bland. Flavor and texture were the most frequently used words for the like and dislike descriptors. For T1, the most frequently used words for liking were flavor, good, and texture (Figure 2) and for disliking the most commonly used words were texture, taste, and flavor. As for the like descriptors, the most commonly used for T2 were flavor, good, and texture, while for dislike descriptors, the most commonly used word was texture. More positive words to describe the quality of the patties were used for T2 than negative words. As for like descriptors, the most commonly used words for T3 were flavor, texture, good, like, and the most frequently used word for disliking was texture. More positive words to describe the quality of the patties were used for T3 than were negative words.(Figure 3,4)

For all vegetable patty samples, more descriptive words were used when the consumer panelists responded to describe liking points of the sample compared to dislike descriptors. Across all the words clouds, texture was most consistently used for describing whether or not a consumer liked a sample except for T1 for the like descriptors.

### 3.4 Color of raw and cooked vegetable patties

Table 5 shows the color of raw and cooked vegetable patties with HMMA. The protein source did not affect the color of raw and cooked patties for lightness (L*, *p* = .09) and cooked patties for yellowness (*p* = .42) compared to C1. However, the protein source significantly affected the redness of raw patties (*p* = .0012) and yellowness (*p* = .0044) and redness in cooked patties (*p* = .05). Redness and yellowness were higher for these samples of raw patties containing pulse proteins compared to the control, and redness was similar for T2 to T1 and T3, but higher for T1 compared to T3. Yellowness was similar for these samples of raw patties to each other containing PLP. Redness was similar for these samples to each other.

**TABLE 5:** Color of raw and cooked, HMMA vegetable patties

Attribute	*P*‐value	C1	T1	T2	T3	^b^RMSE
Raw						
L*	0.09	57.4	56.1	56.6	57.1	0.55
a*	0.001	4.4^c^	6.4^a^	5.8^a^, ^b^	5.5^b^	0.38
b*	0.004	20.0^b^	23.5^a^	23.1^a^	22.8^a^	0.87
Cooked						
L*	0.27	57.5	57.6	55.3	55.8	1.63
a*	0.05	6.8^b^	8.5^a^, ^b^	9.2^a^	8.9^a^	0.96
b*	0.42	22.8	24.7	23.6	24.3	1.49

^a^ Means within a row and effect followed by the same letter are not significantly different (*p* >.05).

^b^ RMSE = root mean square error.

^c^ Recipes for texturization: C1 (control) = soy concentrate and soy isolate, T1, T2, and T3 = pea proteins, lentil proteins, and faba bean proteins, respectively, premixed with a constant ingredient (canola oil and wheat gluten).

### 3.5 Cooking properties and texture of vegetable patties containing HMMA with PLP

Table 6 shows cooking properties (cooking yield and time) and texture (hardness, cohesiveness, and gumminess) of vegetable patties containing HMMA with different PLP. The protein source in vegetable patties containing HMMA significantly (*p* = <0.0001) affected cooking yield and cooking time. Cooking yield was the highest (96.20%) for T3 and lowest for C1 (92.53%) compared to other samples, followed by T3 which was not significantly different from T2 which was like T1. The cooking time of C1 was not significantly different from T1 and T2, but C1 required more cooking time than T3, which was like T2. The more the patties cooked, the more water evaporated and resulted in a decrease in cooking yield.

**TABLE 6:** Cooking yield, cooking time, and texture of cooked HMMA vegetable patties with PLP

Attribute	*P*‐value	C1	T1	T2	T3	^b^RMSE
Cooking parameters						
Cooking yield, %	<0.0001	92.5^c^	93.6^b^	94.1^a^, ^b^	96.2^a^	1.62
Cooking time, min	<0.0001	5.1^a^	4.9^a^	4.6^a^, ^b^	4.1^b^	0.80
TPA						
Hardness, *N*	0.1	67.3	52.0	59.4	57.5	9.05
Cohesiveness	0.002	0.4^a^	0.3^b^	0.3^b^	0.3^b^	0.02
Gumminess	0.009	24.9^a^	16.8^b^	19.6^a^, ^b^	18.2^b^	3.37

^a^ Means within a row and effect followed by the same letter are not significantly different (*p* >.05).

^b^ RMSE = root mean square error.

^c^ Recipes for texturization: C1 (control) = soy concentrate and soy isolate, T1, T2, and T3 = pea proteins, lentil proteins, and faba bean proteins, respectively, premixed with a constant ingredient (canola oil and wheat gluten).

The protein source in vegetable patties containing HMMA did not significantly (*p* = .1) affect hardness but did significantly affected cohesiveness (*p* = .002) and gumminess (*p* = .009). C1 had the highest hardness, cohesiveness, and gumminess compared to other samples except T2 in which gumminess was not significantly different from C1. Other patties containing PLP did not have significantly different cohesiveness and gumminess.

## 4 CONCLUSION

Trained panelists (*n* = 9 or 10) evaluated the flavor and texture of HMMA containing different PLP to compare to C1, a soy‐based HMMA. Bean‐like, salty, sweet, umami, heated oil and cohesiveness of mass were significantly higher for HMMA containing PLP than C1 while soy, green, sweet, cardboardy, musty‐earthy, malt‐like, hardness, and springiness were significantly lower than C1.

Consumer panelists (*n* = 80) conducted a sensory evaluation to evaluate their preferences (cooked appearance, overall, overall flavor, and overall texture) of vegetable patties made with HMMA containing different PLP compared to the control containing soy‐based protein. Different protein sources in vegetable patties did not significantly influence the consumers’ liking of the cooked appearance, overall, and overall flavor except for T3, which had a lower overall liking compared to C1. In contrast, overall texture was lower for vegetable patties containing PLP. The most frequently used words from consumer panelists in response to the open‐ended question about liking or disliking a sample was texture.

The protein source did not affect the color of raw and cooked patties for lightness and cooked patties for yellowness compared to C1. However, the protein source significantly affected the redness and yellowness of raw patties and redness for cooked patties. Cooking yield was higher, and cooking time was lower for vegetable patties containing PLP compared to the control. They did not have significantly different hardness, or significantly lower cohesiveness or gumminess compared C1.

Therefore, PLP can be an alternate source of soy to produce HMMA since consumers scored a similar liking of vegetable proteins containing different PLP. In addition, the cooking yield of the samples containing PLP was higher than C1, and they needed relatively less cooking time. Although the TPA gave lower textural properties in cohesiveness and gumminess for the vegetable patties containing PLP, these proteins might provide a unique combination of attributes and attract consumers.

## CONFLICT OF INTEREST

The authors, whose names are listed above, swear that they have no affiliations with or involvement in any organization or entity with any financial interest (such as honoraria; educational grants; participation in speakers’ bureaus; membership, employment, consultancies, stock ownership, or other equity interest; or expert testimony or patent‐licensing arrangements) or nonfinancial interest (such as personal or professional relationships, affiliations, knowledge or beliefs) in the subject matter or materials discussed in this manuscript.

## STUDIES INVOLVING HUMAN SUBJECTS

Human subjects for this study were approved by the Institutional Review Board for the Protection of Human Subjects in Research (IRB) protocol IRB2017‐0362 M.

## STUDIES INVOLVING ANIMAL OR HUMAN SUBJECTS

Panelist training and testing was approved by the Institutional Review Board for the Protection of Human Subjects in Research (IRB) protocol IRB2017‐0362 M. Eighty consumers recruited through a flyer and email and participated in this sensory test approved by the IRB (IRB2017‐0362 M).

## Data Availability

The datasets generated during and/or analyzed during the current study are available in the Texas A&M University Libraries repository, https://oaktrust.library.tamu.edu/handle/1969.1/173522
